# Physiotherapeutic Interventions for Patients With Rare Genetic Muscle-Wasting Disorders: A Systematic Review and Meta-Analysis

**DOI:** 10.7759/cureus.66349

**Published:** 2024-08-07

**Authors:** Abdullah Alzahrani

**Affiliations:** 1 Department of Health Rehabilitation Sciences, College of Applied Medical Sciences, Shaqra University, Shaqra, SAU

**Keywords:** exercise therapy, motor function, 6-minute walk test, genetic muscle-wasting disorders, physiotherapy

## Abstract

Patients with rare genetic muscle-wasting disorders (MWDs) often experience significant motor function impairments, making effective management strategies crucial for improving their quality of life. This systematic review and meta-analysis aimed to evaluate the impact of physiotherapeutic interventions on motor outcomes in this patient population.

A comprehensive literature search was conducted to identify randomized controlled trials (RCTs) and cohort-based studies that assessed physiotherapeutic interventions in patients with rare genetic MWDs. The primary outcome measure was the 6-minute walk test (6MWT). A random effects model was employed to calculate the mean difference (MD) and 95% confidence interval (CI).

Nine studies were selected for inclusion, and most demonstrated observable improvement in different facets of individuals with MWDs using physiotherapy. The meta-analysis of RCTs showed that physiotherapy statistically improved 6MWT performance (MD: -35.25 meters; 95% CI: -54.14 to -16.37) with low heterogeneity (Tau² = 0.00; Chi² = 0.48, df = 2, P = 0.79; I² = 0%). Similarly, the cohort-based studies demonstrated an overall MD (MD: -10.00; 95% CI: -11.07 to -8.93), with low heterogeneity (Tau² = 0.00; Chi² = 0.01, df = 1, P = 0.94; I² = 0%). Both analyses indicated significant improvements in 6MWT performance (RCTs: Z = 3.66, P = 0.0003; cohort-based: Z = 18.26, P < 0.00001).

Physiotherapeutic interventions significantly enhanced motor function in patients with rare genetic MWDs, as evidenced by improved 6MWT performance. Exercise and intensive physiotherapy programs were particularly effective, although the benefits varied depending on the specific intervention and patient population. These findings support incorporating tailored physiotherapeutic strategies in MWD management to improve motor outcomes and overall quality of life.

## Introduction and background

Rare genetic muscle-wasting disorders (MWDs) are characterized by progressive muscle weakness and deterioration. These disorders present significant challenges in clinical management and treatment options [1-2]. Physiotherapeutic treatments have emerged as a crucial strategy for comprehensively treating individuals suffering from these disorders. However, comprehensive research on the effectiveness and efficiency of physiotherapeutic treatments for this specific group is lacking. Thus, the objective of this systematic review and meta-analysis is to provide a thorough assessment of the body of knowledge on physiotherapeutic treatments for individuals with MWDs [3].

Rare genetic MWDs, such as myotonic dystrophy, spinal muscular atrophy (SMA), and Duchenne muscular dystrophy (DMD), are characterized by a gradual and irreversible decline in muscle strength and function. These disorders frequently arise from genetic abnormalities affecting muscle structure, function, or metabolism. Their atypical nature hinders our comprehensive understanding of these diseases and the development of effective therapeutic strategies [4-5].

Non-pharmacological approaches, such as exercise programs, physical therapy procedures, and rehabilitation techniques, collectively known as physiotherapeutic therapies, have been utilized to manage symptoms and improve the quality of life for individuals with rare genetic MWDs [6]. These therapies aim to enhance functional independence, improve motor performance, increase muscle strength, maintain joint range of motion, prevent complications, and ultimately optimize functional independence [3].

Physical therapy is critical in the multimodal strategy for treating patients with MWDs. Experts recognize treadmill walking as an effective intervention for improving adaptive mechanisms related to coordination, body orientation, and balance control [7]. In this therapeutic approach, the patient assumes an upright posture and bears weight on their lower limbs while engaging in repeated, rhythmic walking motions [8]. Treadmills attract clinicians and researchers because they offer a controlled, continuous environment. Researchers use treadmills to replicate normal walking on solid ground, and in clinical settings, they aim to enhance overground gait [9].

Although specific studies in this population have shown favorable results linked to various physiotherapeutic approaches, a thorough and systematic review of the current evidence is much needed [10-13]. Therefore, this review aims to close this gap by evaluating the existing information on physiotherapeutic therapies for patients with rare genetic MWFs and clarifying the advantages and limitations of these interventions in this population.

## Review

Materials and methods

Eligibility Criteria

Researchers adhered to Preferred Reporting Items for Systematic reviews and Meta-Analyses (PRISMA) standards [14] to ensure a systematic review process. The review scope was defined using the PECO protocol, focusing on individuals diagnosed with rare genetic muscle-wasting disorders (P) undergoing intensive physiotherapy and structured exercise routines (E), compared to standard care without alternative exercise methods (C). Motor function improvement was primarily assessed via the 6-minute walk test (6MWT). Inclusion and exclusion criteria are outlined in Table 1.

**Table 1:** Selection criteria devised for the review FSHD: Facioscapulohumeral muscular dystrophy; SMA: spinal muscular atrophy; DMA: Duchenne muscular dystrophy; HD: Huntington's disease

Criteria	Inclusion	Exclusion
Population	Patients diagnosed with rare genetic muscle-wasting disorders (e.g., FSHD, SMA, DMD, HD)	Patients without a diagnosis of rare genetic muscle-wasting disorders
Intervention	Physiotherapeutic interventions, including structured exercise programs, home-based training, and intensive physiotherapy regimens	Studies focused solely on pharmacological treatments or other non-physiotherapeutic interventions
Comparator	Comparator groups included no intervention, standard care, or alternative exercise modalities	Studies lacking a comparator group or using non-standardized comparators
Outcome	Measures of motor function improvements (primarily 6MWT), other functional assessments, muscle strength parameters, and quality of life measures	Studies not measuring motor function or related outcomes or using non-validated outcome measures
Study design	Randomized controlled trials (RCTs), cohort studies, and other comparative observational studies	Case reports, case series, reviews, and editorials
Language	Studies published in English	Studies published in languages other than English
Publication date	Studies published from inception to June 2024	Studies published outside the predefined publication date range

Search Strategy

We developed a systematic database search strategy using Boolean operators and MeSH keywords described in Table 2. Searches were conducted across PubMed, Cochrane Library, Embase, Web of Science, Scopus, CINAHL, and PEDro.

**Table 2:** Search phrases/terms utilised across the databases FSHD: Facioscapulohumeral muscular dystrophy; SMA: spinal muscular atrophy; DMD: Duchenne muscular dystrophy; HD: Huntington's disease

Database	Search string
PubMed	("physiotherapy" OR "physical therapy" OR "exercise" OR "rehabilitation") AND ("genetic muscle-wasting disorders" OR "facioscapulohumeral dystrophy" OR "spinal muscular atrophy" OR "Duchenne muscular dystrophy" OR "Huntington’s disease")
Cochrane Library	(("physiotherapy" OR "physical therapy" OR "exercise" OR "rehabilitation") AND ("genetic muscle-wasting disorders" OR "FSHD" OR "SMA" OR "DMD" OR "Huntington’s disease"))
Embase	('physiotherapy' OR 'physical therapy' OR 'exercise' OR 'rehabilitation') AND ('genetic muscle-wasting disorder' OR 'facioscapulohumeral muscular dystrophy' OR 'spinal muscular atrophy' OR 'Duchenne muscular dystrophy' OR 'Huntington disease')
Web of Science	TS=("physiotherapy" OR "physical therapy" OR "exercise" OR "rehabilitation") AND TS=("genetic muscle-wasting disorders" OR "facioscapulohumeral dystrophy" OR "spinal muscular atrophy" OR "Duchenne muscular dystrophy" OR "Huntington’s disease")
Scopus	TITLE-ABS-KEY ("physiotherapy" OR "physical therapy" OR "exercise" OR "rehabilitation") AND TITLE-ABS-KEY ("genetic muscle-wasting disorders" OR "facioscapulohumeral dystrophy" OR "spinal muscular atrophy" OR "Duchenne muscular dystrophy" OR "Huntington’s disease")
CINAHL	("physiotherapy" OR "physical therapy" OR "exercise" OR "rehabilitation") AND ("genetic muscle-wasting disorders" OR "FSHD" OR "SMA" OR "DMD" OR "Huntington’s disease")
PEDro	("physiotherapy" OR "physical therapy" OR "exercise" OR "rehabilitation") AND ("genetic muscle-wasting disorder" OR "facioscapulohumeral dystrophy" OR "spinal muscular atrophy" OR "Duchenne muscular dystrophy" OR "Huntington’s disease")

Data Extraction 

Data extraction followed a standardized protocol to minimize bias. Two independent reviewers used a pre-defined extraction form, resolving discrepancies through discussion or consultation with a third reviewer. Extracted data items are detailed in Table 3.

**Table 3:** Data items and characteristics

Serial number	Data items	Characteristics
1	Study identification	This included the title, authors and year of publication
2	Study design	Information on the type of study (e.g., randomized controlled trial, cohort study), sample size, and duration of the study was extracted
3	Population characteristics	Details about the participants, including age, gender, diagnosis of the genetic muscle-wasting disorder, disease severity, and inclusion and exclusion criteria, were collected
4	Intervention details	Comprehensive information on the physiotherapeutic interventions was recorded. This included the type of intervention (e.g., structured exercise programs, home-based training, intensive physiotherapy), duration, frequency, and intensity of the interventions
5	Comparator details	Data on the comparator groups, including no intervention, standard care, or alternative exercise modalities, were documented
6	Outcome measures	The primary outcome was usually the 6-minute walk test (6MWT). Secondary outcomes included other functional assessments, muscle strength parameters, and quality of life measures
7	Results	The main findings of the studies were recorded, focusing on the quantitative data related to the primary and secondary outcomes. This included mean differences, confidence intervals, p-values, and any reported effect sizes
8	Risk of bias assessment	Information related to the assessment of bias within each study was extracted. This included details on randomization, blinding, incomplete outcome data, and selective reporting

Bias Assessment Protocol

Bias was assessed using Cochrane's RoB 2.0 tool [15] and ROBINS-I tool [16], with GRADE method [17] used to evaluate evidence certainty, considering factors like inconsistency, indirectness, imprecision, and publication bias.

Meta-Analysis Protocol

Meta-analysis was conducted using RevMan 5 version 5.4.1 (IBM Corp., USA). 6MWT data from included trials were analyzed using a random-effects model to assess variability (Chi-square test, I² statistic).

Results

Initial Database Search

**Figure 1:**
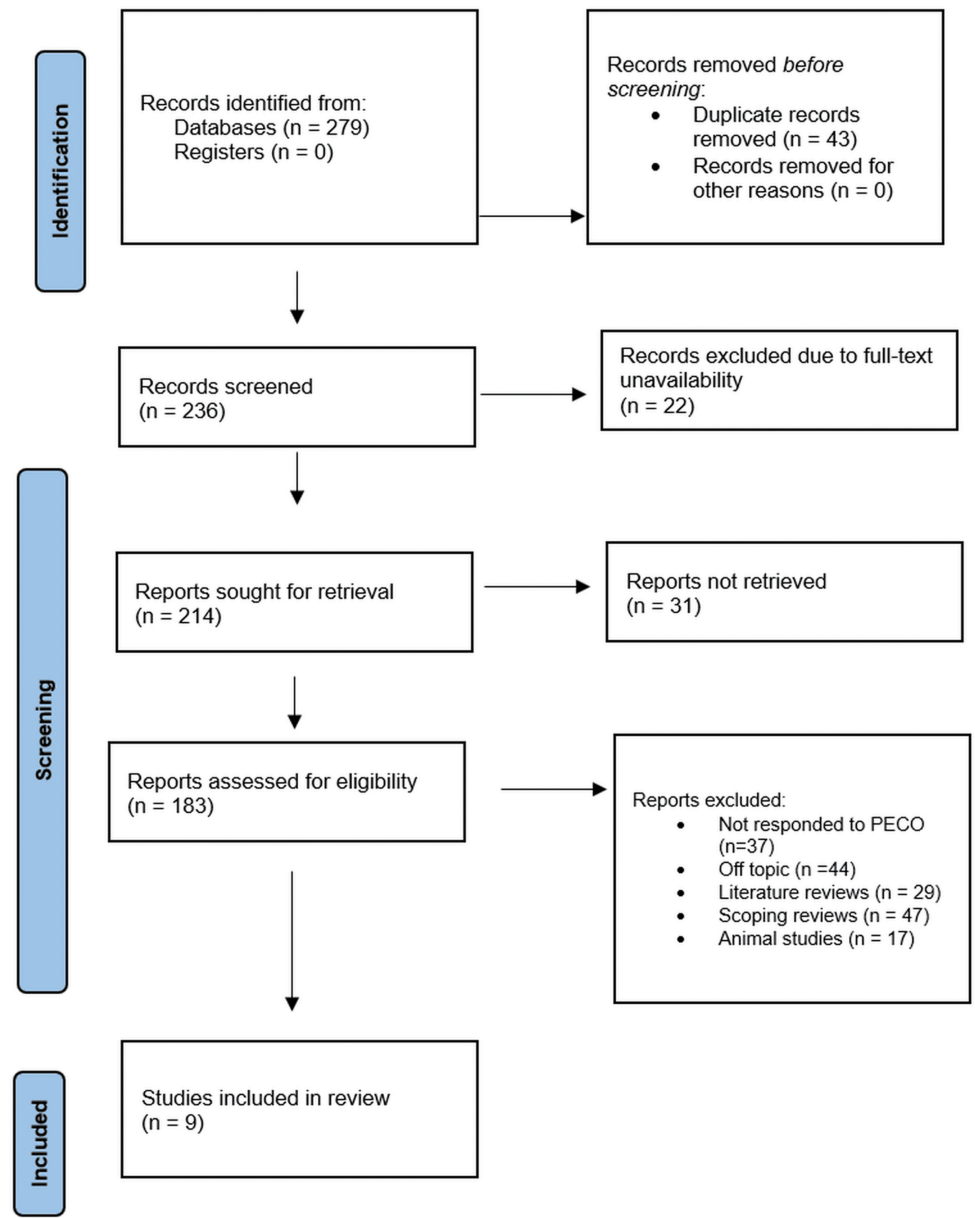
PRIMSA flow chart PRISMA: Preferred Reporting Items for Systematic reviews and Meta-Analyses

Database searches initially yielded 279 records overall (Figure 1) with no additional records found during registration.

Duplicate Records Removal

The original pool had 43 duplicate records removed before the screening process.

Screening Process

Two hundred and thirty-six records in total were screened based on their titles and abstracts. Of the 236 examined records, 22 were eliminated because full-text versions were unavailable. One sought to retrieve the remaining 214 reports. Still, despite best efforts to find them, 31 reports were nowhere to be found.

Eligibility Evaluation

After retrieval, we evaluated the 214 reports for eligibility using pre-defined inclusion and exclusion rules. In this evaluation, 183 reports were omitted for different reasons. These exclusions comprised papers that did not fit the PECO framework (n = 37), reports off-topic (n = 44), literature reviews (n = 29), scoping reviews (n = 47), and animal studies (n = 17). Following this procedure, we included nine papers (Table 4) [18-26].

**Table 4:** Trials included in the review and their observed assessments FSHD: Facioscapulohumeral muscular dystrophy; SMA: spinal muscular atrophy; DMD: Duchenne muscular dystrophy; HD: Huntington's disease; RCT: randomized controlled trial

Author	Year	Sample size	Study design	Disorder assessed	Intervention parameters	Statistics observed	Overall inference drawn
Bankolé et al. [18]	2016	16 FSHD patients	RCT	FSHD	6-month home-based exercise (cycling) 3x/week for 35 mins	VO2 peak (+19%, P=0.002) and MAP improved by week 6; muscle endurance, MVC, walking distance increased; muscle CSA and citrate synthase activity increased	Home-based exercise improves fitness and motor function without compromising muscle tissue
Lessard et al. [19]	2021	All males	Pre-post-test design	Myotonic Dystrophy Type 1	10-week semi-supervised home exercise (sit-to-stand, squat, lunges)	Significant improvement in knee flexor strength; all participants improved in at least two measures	Home-based training is beneficial and well-accepted, reducing healthcare burden
Lewelt et al. [20]	2015	Nine SMA children	Feasibility and safety study	SMA Types II and III	12-week supervised home-based progressive resistance training 3x/week	90% adherence; pain-free sessions; trends in improved strength and motor function	Progressive resistance training is feasible, safe, and potentially beneficial in SMA children
Mirea et al. [21]	2022	Fifty five SMA patients (children/adolescents)	Comparative observational study	SMA	Nusinersen therapy with/without daily physical therapy	Motor skill improvements: Study group (12.66%) vs. control group (3.18%), p < 0.001	Regular physical therapy enhances motor outcomes significantly compared to nusinersen alone
Montes et al. [22]	2015	Fourteen SMA patients (10-48 years)	RCT	SMA	6-month individualized home-based cycling and strengthening	Percent-predicted VO2 max improved 4.9% in 6 months, p=0.036; no change in 6MWT, fatigue, or function	Daily exercise is safe, improves VO2 max, and should be encouraged for ambulatory SMA patients
Piira et al. [23]	2013	Thirty-seven HD patients	Prospective intervention study	HD	One-year multidisciplinary rehabilitation program, including physical exercise, social activities, group sessions, and coordinated health care services	Significant improvements in gait (TUG: -1.32s, 10MWT: +0.27 m/s, 6MWT: +68.71m), balance (BBS: +1.0, p<0.03), physical QoL, anxiety and depression (-3.54 points, p<0.001), and BMI (+0.72 units, p<0.024)	Intensive rehabilitation improves gait, balance, physical QoL, and reduces anxiety and depression in HD patients; cognitive function remained stable except for SDMT (declined by 2.87 points, p<0.05)
Ramberg et al. [24]	2023	Sixty nine patients with various MWDs	Quality improvement study	Various MWDs	Two-week intensive individualized physiotherapy	Significant improvement in gait (10MWT, F8WT: 4-5%) and balance (SLS, MSL: 12-16%), p<0.001	Intensive individualized physiotherapy improves gait and balance in MWD patients
Savcun et al. [25]	2019	Eleven FSHD patients	Observational study	FSHD	Scapular taping during shoulder elevation	No significant change in scapular kinematics (p > 0.05) before and after taping on both sides	Kinesio taping does not significantly alter scapular kinematics during shoulder elevation in FSHD patients
Sherief et al. [26]	2021	Thirty DMD boys (6-10 years)	RCT	DMD	Aerobic exercise: bicycle ergometer vs. treadmill	Significant improvement in walking capacity and balance post-treatment (P < 0.05); greater improvement in treadmill group (P < 0.05)	Treadmill training is more effective than bicycle ergometer in improving walking capacity and balance in DMD children

Assessed Domains of Bias

**Figure 2:**
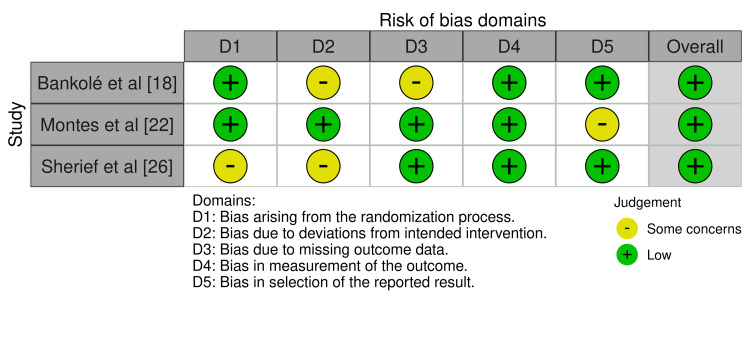
Assessed bias using the RoB 2.0 tool

The bias assessment using the RoB 2.0 method found that Bankolé et al. [18], Montes et al. [22], and Sherief et al. [26] had a relatively low overall risk of bias, particularly about selective reporting and outcome measurement (Figure 2).

**Figure 3:**
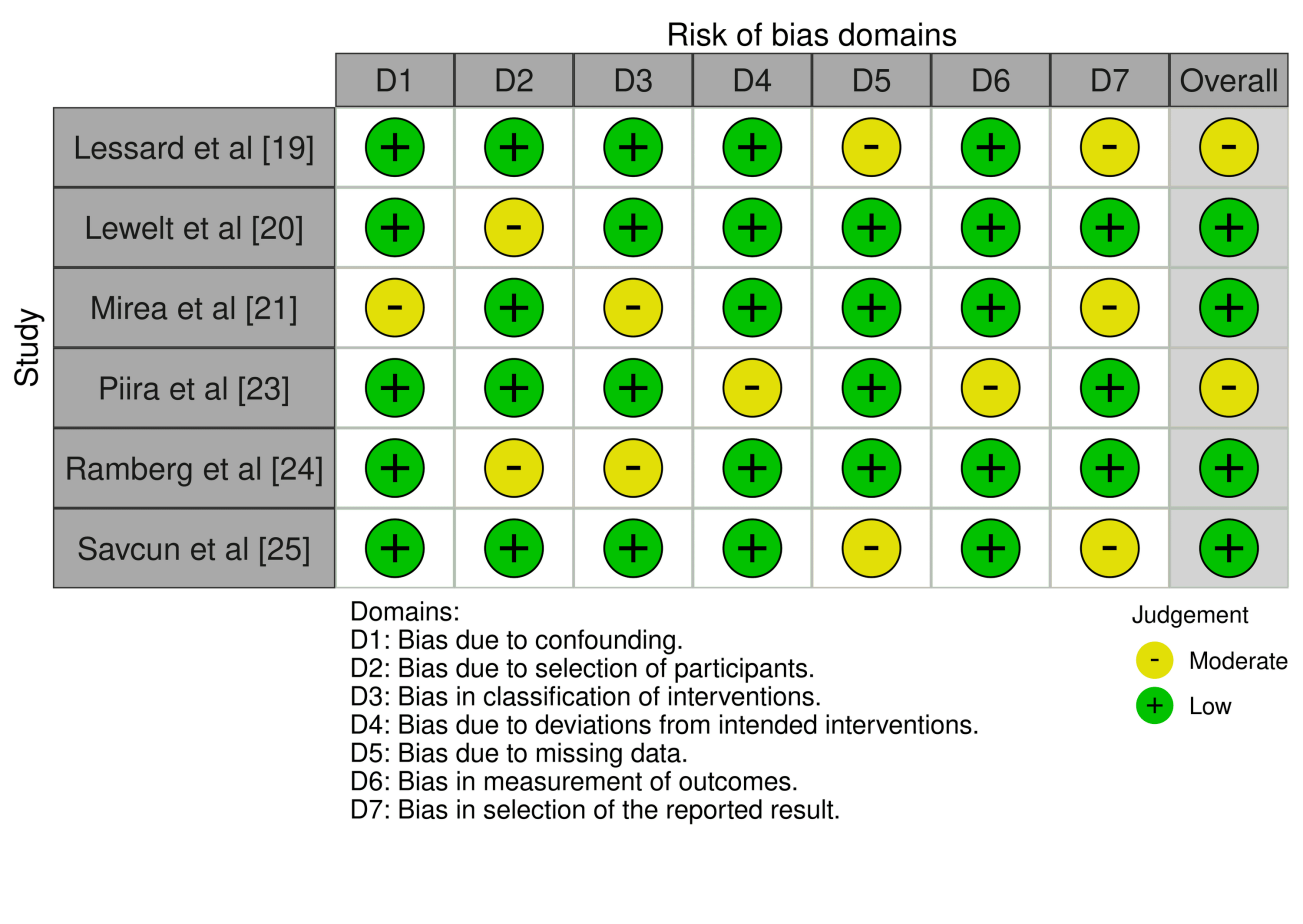
Assessed bias using the ROBINS-I tool

Non-randomized studies have shown increased variability using the ROBINS-I instrument (Figure 3). The minor overall risk shown in the studies conducted by Lessard et al. [19] and Piira et al. [23] can be attributed to confounding factors and missing data. Lewelt et al. [20], Mirea et al. [21], Ramberg et al. [24], and Savcun et al. [25] raised minor issues, mainly about confusion and selection bias, despite the overall low risk.

Study Designs and Sample Sizes Observed

Table 3 presents the observed parameters examined in the physiotherapeutic treatments for MWDs across the trials included in the study [18-26]. Bankolé et al. [18] conducted a six-month home-based cycling program with patients diagnosed with facioscapulohumeral muscular dystrophy (FSHD). The findings demonstrated notable enhancements in mean arterial pressure, muscle endurance, maximum voluntary contraction, walking distance, muscle cross-sectional area, and citrate synthase activity (an increase of 19%, P = 0.002). Lessard et al. [19] implemented a 10-week semi-supervised home exercise program for individuals with DM1, resulting in notable enhancements in knee flexor strength and performance across multiple metrics for all participants. Lewelt et al. [20] conducted a 12-week supervised resistance training program at home for individuals with SMA types II and III. The participants showed a high level of adherence (90%), experienced pain-free sessions, and showed positive trends in both strength and motor function improvement. In their study, Mirea et al. [21] compared nusinersen therapy with and without daily physical therapy for SMA. They observed that the study group exhibited a substantially better improvement in motor skills (12.66%) compared to the control group (3.18%), with a p-value of less than 0.001. Montes et al. [22] assessed the effectiveness of a personalized home-based exercise program for SMA over six months. They observed a 4.9% enhancement in percent-predicted VO2 max (p = 0.036) but no alterations in 6MWT, tiredness, or function. Piira et al. [23] implemented a comprehensive rehabilitation program for Huntington's disease (HD) that lasted for one year. They observed noteworthy enhancements in gait, balance, physical quality of life, levels of anxiety and despair, and body mass index (BMI). Ramberg et al. [24] conducted a two-week intense physiotherapy intervention for individuals with multiple walking disabilities and observed noteworthy enhancements in gait and balance (p<0.001). Savcun et al. [25] conducted a study on the effects of scapular taping for FSHD and found that there were no significant alterations in scapular kinematics (p > 0.05). In their study, Sherief et al. [26] compared bicycle ergometers and treadmill training for individuals with DMD. The results demonstrated considerable enhancements in walking capacity and balance, with the treadmill group seeing even more significant benefits (P < 0.05).

Meta-Analysis Observations

**Figure 4:**
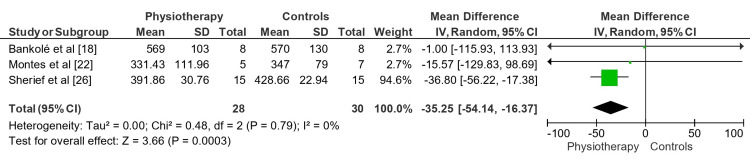
Impact of physiotherapeutic interventions on rare MWDs across the RCTs included in the review MWDs: Muscle-wasting disorders; RCTs: randomized controlled trials

Figure 4 shows the mean difference (MD) depicting the effect of physiotherapy versus controls on the 6MWT for MWDs across the included RCTs. The overall mean difference was -35.25 (95% CI: -54.14 to -16.37). Consistent results across trials were shown by low heterogeneity (Tau² = 0.00; Chi² = 0.48, df = 2, P = 0.79; III = 0%). The overall effect test (Z= 3.66, p=0.0003) indicates that physiotherapy significantly improved 6MWT performance in patients with MWDs compared to control.

**Figure 5:**
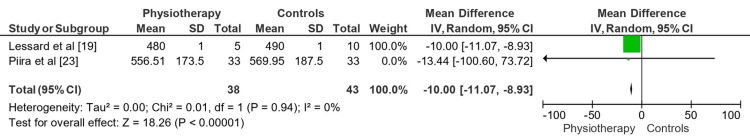
Impact of physiotherapeutic interventions on rare MWDs across the cohort-based trials included in the review MWDs: Muscle-wasting disorders

Figure 5 illustrates the MD showing the impact of physiotherapy versus controls on the 6MWT for MWDs across the included cohort-based studies. With an overall MD of -10.00 meters (95% CI: -11.07 to -8.93), indicating consistent results throughout the trials, the heterogeneity was modest (Tau² = 0.00; Chi² = 0.01, df = 1, P = 0.94; III = 0%).

The overall effect test revealed that physiotherapy statistically improved 6MWT performance, similar to Figure 4 (Z = 18.26, P = 0.00001).

Certainty Bias Assessed

Based on the GRADE evaluation (Table 5), both cohort/observational studies and RCTs provided moderate confidence in the findings. Physiotherapy including home-based and intensive programs markedly improved motor performance and fitness without sacrificing muscle tissue, as was common throughout all trials. There was remarkable consistency in the research, and comparable positive results were mentioned. Since the investigations directly tackled the research question, the evidence remained direct. Precision was likewise vital; tight confidence ranges show consistent approximations of effect sizes. No other element, including publication bias or strong effects, justified a change in the confidence level. With modest certainty, the data generally supports the positive effect of physiotherapy on motor outcomes in this patient population.

**Table 5:** GRADE assessment observations RCTs: Randomized controlled trials

Study design	Number of studies	Common finding	Risk of bias	Consistency	Directness	Precision	Other factors	Certainty
RCTs	4	Physiotherapy interventions improve motor function and fitness.	Low to moderate	Consistent	Direct	Precise	None	Moderate
Cohort and observational studies	5	Home-based and intensive physiotherapy programs are beneficial for muscle strength and motor function.	Low to moderate	Consistent	Direct	Precise	None	Moderate

Discussion

Most of the included trials [18-24] collectively confirmed the efficacy of exercise and intensive physiotherapy interventions. Sherief et al. [26] emphasized the superior efficiency of treadmill exercise over bicycle ergometers for children with DMD. In contrast, Savcun et al. [25] observed no appreciable advantages from scapular taping, highlighting variations in intervention results.

Bankolé et al. [18] and Sherief et al. [26] have demonstrated that structured exercise programs significantly improve motor ability. Sherief et al. [26] reported more significant improvements in walking ability and balance in children with DMD who underwent treadmill training than those using bicycle ergometers. Conversely, Bankolé et al. [18] reported enhancements in fitness and muscle function in FSHD patients. Lessard et al. [19] and Lewelt et al. [20] recommended home-based fitness programs. Lewelt et al. [20] found that SMA children improved strength and motor function, while Lessard et al. [19] revealed significant increases in knee flexor strength in DM1 patients. Both studies underscored the efficacy of home-based training in enhancing muscular strength.

Studies by Mirea et al. [21] and Montes et al. [22] have shown that regular physical exercise or therapy significantly improves motor outcomes in people with SMA. Montes et al. [22] observed increased VO2 max but no changes in functional measures such as 6MWT. On the other hand, Mirea et al. [21] revealed that physical therapy with nusinersen led to more significant improvements in motor skills.

Piira et al. [23] and Ramberg et al. [24] found notable gains in gait and balance through rigorous physiotherapy. While Ramberg et al. [24] focused on gait and balance improvements in MWD patients, Piira et al. [23] also demonstrated improvements in physical QoL, anxiety, depression, and BMI in HD patients. Both studies validated the effectiveness of intense physical treatment. However, unlike earlier research, Savcun et al. [25] found no appreciable changes in scapular kinematics with scapular taping in FSHD patients, contrasting with favorable results reported from exercise and rigorous treatment.

Research on how physiotherapeutic training programs specifically impact lower-limb muscle strength and related activities in the target group is limited [27]. Lindeman et al. [28] conducted a study using strength weights [27-28], which found no statistically significant increases in lower limb muscular strength and mobility-related activities following a 24-week home-based training program. However, their study did find a notable increase in muscle endurance. Other studies have demonstrated improvements in maximum muscular strength [29-32] and the performance of several mobility-related activities [29,32-35]. These methodological variations complicate comparisons across studies.

Several reviews [36-40] conducted on objectives similar to ours have shown mixed results regarding the efficacy of physiotherapy in alleviating signs and symptoms of MWDs. Fritz et al. [36] were the first to demonstrate that exercise and physical activity can benefit people with HD. Our study confirmed that physiotherapy improves motor function in people with rare genetic MWDs. Both evaluations noted changes in motor performance, gait speed, and balance, highlighting variations in intervention strategies and outcome measures as limiting factors. Similarly, our results resonate with Graham et al. [1], who emphasized the role of exercise in preventing or reducing muscle wasting through molecular and physiological changes. Our investigation showed that physiotherapy interventions, including home-based and intense programs, significantly enhanced motor performance and muscle strength, consistent with the broader benefits of exercise highlighted by Graham et al. [1].

However, the results of Bartels et al. [37] and Jones et al. [11] showed different trends. Bartels et al. [37] examined SMA type 3 patients and found no significant differences in walking distance or VO2 max between the training and regular care groups. This contrasts with our study, which found improvements in 6MWT performance and motor function. Differences in intervention adherence and the limited sample size in Bartels et al. [37] may explain this disparity, contributing to the uncertainty of the evidence.

Jones et al. [11] found that many included studies did not report usable physical activity outcomes, and most had a high or unclear risk of bias. In contrast, our study consistently showed improvements in motor function and physical performance from physiotherapy interventions. This contrasts with our results, where the included studies generally showed low to moderate bias risks and demonstrated the benefits of physiotherapy.

Consistent with Leone et al. [38], our study showed that in patients with muscular dystrophy, strength training and other exercise therapies enhanced motor function, perceived exertion, balance, gait, and overall well-being. Emphasizing its safety and efficacy in improving physical function and quality of life, both studies advocated integrating strength training into MD treatment plans. Similarly, in patients with MWDs, Silva et al. [39] demonstrated that low- to moderate-intensity aerobic exercise enhanced mobility, self-care, and social interaction. Like Silva et al. [39], our study highlighted the beneficial effects of home-based and controlled exercise programs on motor function and physical performance. Both studies underscored the role of physical activity in improving functional outcomes and increasing engagement in daily activities.

However, our results diverged from those of Pedersen et al. [40]. While Pedersen et al. [40] focused on the effects of strength training on patients with polyneuropathy and reported gains in strength, they highlighted the sparse evidence due to poor methodological quality in the included studies. Periodic improvements were observed in motor performance and athletic ability. Although our review included a more robust collection of evidence supporting the efficacy of physiotherapy interventions, Leone et al. [38] and Silva et al. [39] noted more significant bias risks and fluctuating study quality. Additionally, specific therapies, such as vibratory proprioceptive aids and neuromuscular electrical stimulation, were not addressed in our analysis, as noted by Leone et al. [38].

Limitations

Our observations from this review are subject to some limitations. Firstly, the heterogeneity among patient groups and diseases studied may have influenced outcomes, complicating generalizations to all MWDs. Moreover, the relatively small sample sizes in various studies could have limited the statistical power and validity of our conclusions. Additionally, focusing solely on the 6MWT may have overlooked other significant improvements in functional and quality-of-life measures. These limitations should be considered in future studies to ensure more comprehensive and robust evidence.

Clinical Recommendations

Findings from our review suggest several recommendations for using exercise and physiotherapy interventions in individuals with genetically rare MWDs, including structured exercise programs, regular physical activities, and individualized approaches. Our research indicates that individuals with muscle-wasting diseases can significantly improve physical performance and motor ability through structured exercise programs. To optimize outcomes, these interventions should be tailored to each patient's unique conditions and needs. For example, studies suggest that treadmill exercise is particularly effective for individuals with DMD, enhancing balance and walking capacity more effectively than bicycle exercise. Therefore, integrating treadmill exercise into rehabilitation programs for individuals with DMD could yield substantial benefits. Consistently, research demonstrates that individuals with muscle-wasting diseases experience improved motor outcomes from regular physical activity, including enhanced gait, balance, physical quality of life, and psychological well-being. Hence, integrating these interventions into standard care can be highly beneficial. Adopting an individualized approach is crucial in selecting the appropriate exercise and physiotherapy interventions, considering the varying outcomes reported across different trials. To optimize treatment success, it is essential to tailor interventions to the unique requirements and abilities of each individual.

## Conclusions

Our review concluded that physiotherapeutic therapies significantly enhance motor abilities in patients with rare MWDs. The trials in our analysis demonstrated that rigorous physiotherapy and organized exercise programs, particularly those conducted at home, successfully improved outcomes, including performance on the 6MWT. However, variations in patient demographics, intervention strategies, and study designs underscore the need for additional research to optimize and standardize these therapeutic approaches. These findings suggest that specific physiotherapy should be integrated into the care strategies for these patients to enhance functional capacity and improve quality of life.
